# RNA-sequencing Reveals Altered Gene Expression in the Ventromedial Hypothalamus Following Predator Odor Exposure

**DOI:** 10.12688/f1000research.152034.1

**Published:** 2024-06-17

**Authors:** Ashely Shemery, Megan Gibson, Erin Gorrell, Diamond Daniel, Helen Piontkivska, Colleen M Novak

**Affiliations:** 1 School of Biomedical Sciences, Kent State University, Kent, Ohio, 44242, USA; 2 Department of Biological Sciences, Kent State University, Kent, Ohio, 44242, USA; 3 Brain Health Research Institute, Kent State University, Kent, Ohio, 44242, USA

**Keywords:** Predator threat, skeletal muscle, thermogenesis, corticosterone, sirtuin, BDNF, SF-1

## Abstract

**Background:**

Physical activity is the second largest contributor to our total daily energy expenditure (EE). Uncovering ways to increase EE during activity could yield new approaches to treat obesity. The ventromedial hypothalamus (VMH) regulates body weight by modulating muscle metabolism and sympathetic nervous system (SNS) activity. The VMH also mediates behavioral responses to predator threat. While the VMH is a potential mediator of metabolic responses to predator threat, the mechanisms are unknown.

**Methods and Results:**

Exposing rats to predator odor (PO) exposure causes a rapid increase in skeletal muscle thermogenesis that peaks between 20-30 min and dissipates to baseline by 4 hr. This thermogenic response is associated with weight loss and increased EE even when controlling for physical activity. To probe potential targets of PO-induced metabolic responses in the VMH, we first performed qPCR for genes known to be involved in brain regulation of muscle metabolism using VMH samples from rats exposed to PO or control odor for either 30 min or 4 hr. Next, to uncover novel, relevant genes, we performed RNA-sequencing on VMH samples of rats exposed to either PO or control odor for 20 min. qPCR results show that after 4 hr of PO exposure, *Bdnf* and *Sirt1* expression were increased. RNA-sequencing analyses further identified 245 differentially expressed genes (DEGs) that showed at least 1.5-fold change in expression due to PO exposure. Functional and Gene Ontology annotation showed that pathways related to immune response, oxidative stress, and synaptic plasticity were overrepresented among these DEGs.

**Discussion:**

Taken together, these findings suggest that acute PO exposure induces both rapid and delayed changes in VMH gene expression that likely have downstream metabolic consequences.

## 1. Introduction

Non-exercise activity thermogenesis (NEAT) is the strongest predictor of resistance to weight gain with overfeeding, making it a promising target for obesity treatments ( Levine et al., 1999). Understanding the cellular and molecular mechanisms underlying brain regulation of energy expenditure (EE) is critical when exploring ways to manipulate NEAT. The ventromedial hypothalamus (VMH) plays a necessary role in energy homeostasis through its regulation of autonomic nervous system activity and its complex connections with other brain regions involved in feeding and thermoregulation ( King, 2006). Activation of the VMH induces sympathetic nervous system (SNS) drive to white adipose tissue (WAT), brown adipose tissue (BAT), and skeletal muscle, which was associated with increased thermogenesis in BAT and skeletal muscle ( Gavini et al., 2016). Additionally, the VMH mediates multiple behavioral responses including aggression ( Falkner et al., 2016; Hashikawa et al., 2017), fear responses and memories ( Silva et al., 2016), and aversion to predator odor ( Perez-Gomez et al., 2015). Though not fully understood, the processing of both metabolic signals and predator threat by the VMH reflects the shared importance of SNS outflow in both systems.

Central regulation of BAT thermogenesis and WAT browning have been heavily investigated for their potential to increase EE in small rodents ( Morrison et al., 2014). Though far less investigated, skeletal muscle has a substantial capacity for increasing EE and is clinically relevant as it makes up 40% of total human body mass and accounts for 20-30% of total resting oxygen uptake ( Zurlo et al., 1990; Janssen et al., 2000; van den Berg et al., 2011). Ferret predator odor (PO) exposure to rats causes a rapid increase in skeletal muscle thermogenesis, likely due to SNS activation, that peaks between 20-30 min and dissipates over the course of a few hours ( Gorrell et al., 2020). Chronic PO exposure for 3 weeks decreases body weight without changing food intake, physical activity, or BAT thermogenesis in mice ( Genne-Bacon et al., 2016), suggesting potential involvement of skeletal muscle. As a dual processor of metabolic and predator threat responses, the VMH is a likely mediator of PO-induced skeletal muscle thermogenesis, though the underlying cellular and molecular mechanisms have remained unexplored.

In the brain, expression of the transcription factor steroidogenic factor 1 (SF-1) is restricted to the VMH, where it is required for VMH development and vitality as well as normal energy balance and response to predator threat ( Ikeda et al., 1995; Shinoda et al., 1995; Kunwar et al., 2015). The VMH, including the SF-1 cell population found there, regulates energy balance by engaging SNS outflow to regulate peripheral tissue metabolism and counteract hypoglycemia ( King, 2006). Importantly, increased VMH SF-1 expression improves skeletal muscle metabolism and is associated with increased SNS activation ( Kim et al., 2012). In addition to SF-1, at least two other genes in the VMH may be critical in SNS-driven skeletal muscle metabolism, namely *Bdnf* and sirtuin 1 ( *Sirt1*). Within the VMH, BDNF is positively associated with and is a downstream target of SF-1 ( Tran et al., 2006). VMH BDNF microinjections activate the SNS and amplify EE by increasing physical activity and resting metabolic rate in the absence of BAT activation ( Wang et al., 2010), suggesting potential contribution from skeletal muscle. SIRT1 is a metabolic sensor protein expressed in many tissues where it regulates metabolism in response in nutrient availability ( Imai et al., 2000; Ramadori et al., 2008). Over- and under-expression of SIRT1 in VMH SF-1 neurons reveals that SIRT1 protects against dietary obesity by regulating EE as well as skeletal muscle glucose uptake ( Ramadori et al., 2011). Moreover, SF-1, BDNF, and SIRT1 expression levels in the brain are all associated with psychological stressors, making these genes possible candidates involved in VMH regulation of skeletal muscle thermogenesis following PO exposure ( Rage et al., 2002; Kunwar et al., 2015; Yu et al., 2018). However, although PO induces c- *fos* in the VMH ( Masini et al., 2005), there is virtually no information regarding the cellular and molecular alterations that follow. Here, we explored changes in VMH expression of *Sf-1*, *Bdnf*, and *Sirt1* in response to PO exposure at time points with thermogenic relevance to the predator-threat response. Additionally, we performed RNA-sequencing to explore transcriptome-wide changes in VMH gene expression after acute PO exposure. Collectively, our results suggest PO causes rapid and delayed transcriptional changes in the VMH that are associated with inflammation, oxidative stress, and synaptic plasticity.

## 2. Methods

### 2.1 Animals

All experiments used Sprague-Dawley rats (Envigo) individually housed in a temperature-controlled room (24°C ± 1°C) on a 12/12 hr light/dark cycle with lights on at 7:00AM and light off at 7:00PM EST. Rats were given free access to both water and standard laboratory chow (5P00 Prolab RMH 3000, LabDiet, St. Louis, MO, USA). To verify the time-course of the muscle thermogenic response, we used 7 rats (3 female, 4 male). For RNA-sequencing, we used 6 male rats. For qPCR and corticosterone assays, we used 24 male rats. All procedures were approved by Kent State University Institutional Animal Care and Use Committee; sample sizes were determined based on prior effect sizes ( Gorrell et al., 2020), with random assignment to experimental condition. Exposure to odor precluded blinding to experimental group or condition. Efforts were made to ameliorate suffering and minimize distress of animals using analgesics before and after surgical procedures, anesthesia, and habituation prior to exposure to potentially stressful contexts.

### 2.2 Surgery

Changes in skeletal muscle temperature in response to predator-odor or control-odor stimuli were measured using surgically implanted temperature transponders (implantable programmable temperature transponder-300; IPTT-300, BioMedic Data Systems, Seaford, DE; calibrated range 32°-43°C); transponders were used that had showed high correlation compared with water-bath temperature (R ^2^ ≥ 0.97) as well as *in vivo* with rectal temperature in the physiological range (R ^2^ ≥ 0.97) ( Wacker et al., 2012). Transponders are 14 mm long and 2 mm in diameter, sufficiently small to surgically implant into the gastrocnemius muscle group bilaterally in rats. Transponders were implanted under surgical anesthesia, with 5% isoflurane for induction followed by 2-3% for maintenance. Animals were given 1 week to recover.

### 2.3 Odor exposure and tissue collection

Control odor or predator odor exposure was achieved by dropping a fragment (1-2” × 2”) of a clean towel or an identical towel that had been used as bedding for ferrets ( *Mustela putorius furo*) for 2 weeks (Marshall BioResources, North Rose, NY) into the rat’s home cage. All animals were habituated to handling as well as daily exposure to control odor in their home cages for 2 weeks prior to experimental odor exposure. For the thermogenic time course experiment, animals were exposed to both PO and control odor (identical towels without contact with ferrets; Marshall BioResources), each on separate experimental days separated by 1 week. Due to potential odor contamination, we declined to counterbalance odor exposure, though order effects have previously been ruled out as a contributor to PO-induced thermogenesis ( Gorrell et al., 2020). Thus, to remain consistent with habituation, all animals were exposed to control odor first. At 3 hrs after lights-on, baseline temperatures were measured using a transponder reader (DAS-7007S; IPTT-300, BioMedic Data Systems, Seaford, DE) to manually retrieve transponder temperature data. To minimize human interference during measurements, animal cages are placed on PVC stands (122 cm × 30 cm × 30 cm) that expose the underside of the cage. The transponder reader can rapidly, accurately, and reliably measure temperature through the cage bottom. Following odor exposure, temperatures were measured at defined timepoints for 4 hr following odor exposure.

For RNA-sequencing, animals were exposed to either control odor (n = 3) or predator odor (n = 3), as described above, for 20 minutes. Animals were then anesthetized via 5% isoflurane inhalation and rapidly decapitated using a guillotine. Brains were dissected out then flash frozen in 10-20 ml isopentane (2-methylbutane; MilliporeSigma #320404) chilled on dry ice. Bilateral micropunches of the VMH were collected. Briefly, brains were coronally sectioned using a cryostat up to the start of the VMH. A micropunching tool 1mm in diameter and 1.5mm in length was inserted into the VMH and samples were ejected into microcentrifuge tubes. Samples were stored in a freezer at -80°C until processing.

For qPCR and corticosterone assays, animals were exposed, as described above, to either control odor for 30 minutes (n = 6) or 4 hours (n = 6), or predator odor for 30 minutes (n = 6) or 4 hours (n = 6). Animals were then anesthetized via isoflurane inhalation and rapidly decapitated using a guillotine. Brains were dissected out and flash frozen in isopentane chilled on dry ice. Trunk blood was collected in 4mL BD Vacutainer™ Plastic Blood Collection Tubes with K2 EDTA using a freshly cleaned plastic funnel. Tubes were inverted to mix then centrifuged at 2,000 × g for 15 minutes at 4°C to separate plasma. Plasma was pipetted into 1.5mL microtubes and stored in the -80 until needed.

### 2.4 RNA sequencing

2.4.1 *RNA isolation, purification and quality check*

To isolate and purify high quality RNA from small VMH micropunches, we performed a technique combining the use of TRIzol and silica-based columns. Micropunches were sonicated in 250 uL of TRIzol™ Reagent (Thermofisher Scientific, Waltham, MA). 50 uL of chloroform was added to homogenate and incubated at room temperature for 5 minutes to facilitate phase separation. Samples were centrifuged at 12,000 × g for 15 minutes at 10°C. The aqueous layer (~100 uL) was pipetted into a fresh microcentrifuge tube and mixed with an equal volume of ice-cold 70% ethanol to precipitate mRNA. The mixture was then pipetted onto a silica-based column membrane (Invitrogen™ PureLink™ RNA Mini Kit, Thermofisher Scientific, Waltham, MA). Samples were centrifuged at 12,000 × g for 30 seconds at 10°C and the supernatant was discarded. Samples were washed as directed by kit instructions. RNA was eluted into a fresh collection tube with 30 uL of nuclease-free water. RNA concentration and quality were assessed using a spectrophotometer. All samples had 80-110 ng/uL, a 260/280 ratio of 2.0-2.1, and a 260/230 ratio of 1.8-2.0. Samples were stored in a freezer at -80°C until processing.

2.4.2 *RNA library preparation*

RNA samples were shipped to Novogene Bioinformatics Technology Company, Ltd. (Sacramento, CA) for sequencing. Before processing, RNA samples passed an integrity and purity assessment via the Qubit ^®^ Fluorometer (Invitrogen, ThermoFisher, Waltham, MA) and Agilent Bioanalyzer ^®^ RNA 6000 Nano/Pico Chip (Agilent Technologies, Santa Clara, CA) with an RNA Integrity Number of ≥ 7. RNA sample libraries were prepared using the NEBNext Ultra II RNA Library Prep Kit for Illumina (New England Biolabs, Ipswich, MA). Briefly, poly-A mRNA was isolated and purified from total RNA before being fragmented and primed with random primers. Following double-stranded cDNA synthesis, ends were repaired and tagged for adaptor ligation. Finally, 250-300bp fragments were isolated and enriched by PCR.

2.4.3 *RNA library quality check*

High-quality RNA library yield and quality were assessed and confirmed using a Bioanalyzer Agilent DNA 1000 Chip (Agilent Technologies, Santa Clara, CA). Sample libraries were sequenced by the Illumina HiSeq 4000 system using CASAVA v1.8 software (Illumina Biotechnology Co., San Diego, CA) with a paired-end 150bp sequencing strategy and sequencing depth of ~5 million reads. An extensive data quality assessment confirmed all sample libraries yielded between 4.6-5.8 million clean reads, 6.9-8.8G clean reads, a base calling error rate of 0.03%, a Q30 score of >93%, and a GC content of 50%.

2.4.4 *Mapping reads to rat genome*

Raw reads were filtered to remove reads containing adapters, reads containing poly-N, and low quality reads. Clean reads were mapped to the Ensembl *Rattus norvegicus* Rnor_5.0 version 79 reference genome using the hierarchical indexing for spliced alignment of transcripts 2 (HISAT2) algorithm v2.1.0-beta ( Kim et al., 2014; Kim et al., 2015; Cunningham et al., 2019). Briefly, the algorithm identifies all represented splice sites. Then, it aligns reads to a single exon of the genome. These reads are segmented and mapped to the adjacent exons. Reads are further segmented and mapped to several exons. A quality assessment revealed that all sample libraries achieved a mapping rate of ~95%, with approximately ~6% of reads mapping to multiple areas of the genome.

2.4.5 *Gene expression quantification and quality check*

HTSeq v0.6.1 was used to count reads mapped to each gene or exon using union mode where each gene is the union of all its exons ( Anders et al., 2015). To normalize mapped read counts, gene expression quantification is expressed in fragments per kilobase of transcript sequence per million base pairs sequenced (FPKM) ( Mortazavi et al., 2008). RNA-sequencing requires biological replicates to ensure accuracy ( Liu et al., 2014). Here, we have 3 replicates per group. Pearson’s correlation coefficient analysis confirms that the genes expressed in each of the control group replicates (r ^2^ = 0.98) as well as the experimental group replicates (r ^2^ = 0.98) are highly correlated indicating minimal intragroup variance.

2.4.6 *Differential gene expression analysis*

Differentially expressed genes were analyzed for statistical significance using DESeq2 v1.38.3 ( Love et al., 2014) using HTSeq counts. The Benjamini-Hochberg procedure was used to control the false discovery rate (FDR) ( Benjamini and Hochberg, 1995). Adjusted p value (padj) cut-off of 0.05 and a fold change of 1.5 were used to identify 245 differentially expressed genes (Table S1). A relatively relaxed fold-change cut-off was used to ensure the capture of a broader set of potentially relevant DEGs.

2.4.7 *Functional annotation, pathway and gene ontology (GO) enrichment analysis*

We further focused on 186 out of 245 DEGs that corresponded to protein-coding genes (Table S1). Functional annotation and protein-protein interaction analysis was performed using STRING ver. 12.0 ( Szklarczyk et al., 2023), followed by annotation and pathway enrichment analysis using ClueGO plug-in v2.5.9 ( Bindea et al., 2009) for Cytoscape ( Shannon et al., 2003). We focused on KEGG, Molecular Function and Immune System Process ontologies in ClueGO, using two-sided hypergeometric test with Benjamini-Hochkins correction for multiple tests. To be considered a cluster, the cut-off of at least 3 genes representing at least 4% of all genes in that category must be present. The leading group terms (shown with the largest circles) are based on the largest number of genes in that category (see also Table S2). Additional Gene Ontology annotations and enrichment analyses were performed using DAVID (The Database for Annotation, Visualization and Integrated Discovery, ver. v2023q3) ( Sherman et al., 2022) and ShinyGO 0.77 ( Ge et al., 2020), with respective FDR cut-offs of 0.05.

2.4.9 *Accession to RNA-seq data*

Data are deposited in NCBI GEO with accession number GSE142617. To review the GEO accession, see https://www.ncbi.nlm.nih.gov/geo/query/acc.cgi?acc=GSE142617.

### 2.5 Corticosterone enzyme-linked immunosorbent assay

Plasma corticosterone was assayed using the Enzo corticosterone ELISA kit (#AD1-900-097, Enzo Life Sciences, Farmingdale, NY). Thawed plasma samples were diluted 1:25 by pipetting 10 uL of plasma sample into microtubes containing 240 uL of nuclease-free water and mixing. To dissociate corticosterone from carrier proteins, samples were incubated in a water bath at 70°C for 1 hr; we found this to be more effective than the steroid displacement technique recommended in the kit instructions. With this exception, the rest of the assay was carried out following kit instructions.

### 2.6 Quantitative real-time PCR analysis

Approximately 160 ng of isolated RNA was reversed transcribed using the High-Capacity cDNA Reverse Transcription Kit (Applied Biosystems, Cheshire, UK). The target cDNA was amplified by PCR. All qPCR assays were carried out in triplicate using the Brilliant III Ultra-Fast QPCR Master Mix (Agilent Technologies; Santa Clara, CA) and using PrimeTime Gene Expression Probes (IDT DNA Technologies). The assay identification numbers for each gene are: *Nr5a1* (Rn.PT.58.33726779), *Sirt1* (Rn.PT.58.44487135), *Bdnf* (Rn.PT.58.13660615), *Gapdh* (Rn.PT.39a.11180736.g). The cDNA quantities were normalized by using the housekeeping gene *Gapdh* as a reference and the Δct method ( Schmittgen and Livak, 2008) was used to analyze relative gene expression. Results are displayed as a percentage of baseline.

### 2.7 Statistics

All data are expressed as the mean ± SEM. Statistical analyses were performed using IBM ^®^ SPSS ^®^ Statistics software version 26 ( https://www.ibm.com/support/pages/spss-statistics-v26-now-available). Figures were created using Systat Software Inc. SigmaPlot version 14.0 ( https://grafiti.com/download-sigmaplot-software/). When applicable, normality distribution was tested with the Shapiro-Wilk normality test. For the thermogenic time course following odor exposure experiment, differences between PO and control odor exposure were analyzed using a 2-way repeated measures ANOVA (odor: PO or control odor; time: 15 time points) followed by Bonferroni post-hoc tests ( Table 1). Post-hoc paired samples t-tests were performed following significant main effects and interactions. Differences in plasma corticosterone levels and VMH expression of *Sirt1*, *Bdnf*, and *Sf1* were analyzed using 2-way ANOVAs (odor, PO or control odor; time, 30 min or 4 hr). RNA-sequencing data were performed as described above. The level of significance was set at *p* ≤ 0.05.

**Table 1.** Statistical analyses for data included in Figures 1- 3.

	Comparison	Data Structure	Type of test	α Level
a	Raw temperature change in gastrocnemius muscle following PO vs control odor exposure	Normally distributed	2-way repeated measures ANOVA, *post hoc* Bonferroni correction Paired samples t-tests (N = 7)	*p* < 0.05
b	Raw temperature change from baseline in gastrocnemius muscle following PO vs control odor exposure	Normally distributed	2-way repeated measures ANOVA, *post hoc* Bonferroni correction Paired samples t-tests (N = 7)	*p* < 0.05
c	Plasma corticosterone levels following PO vs control odor after either 30 min or 4 hr	Normally distributed	2-way ANOVA, *post hoc* Paired samples t-tests (n = 6/group, total N = 24)	*p* < 0.05
d	*Sirt1*, *BDNF*, and *Sf1* mRNA expression levels in the VMH following either PO or control odor exposure after either 30 min or 4 hr	Normally distributed	2-way ANOVA, *post hoc* Paired samples t-tests (n = 6/group, total N = 24)	*p* < 0.05

## 3. Results

### 3.1 Predator odor exposure rapidly induces sustained skeletal muscle thermogenesis

Gorrell et al. (2020) reported that PO exposure increased muscle thermogenesis, peaking between 15-30 min, but did not observe its full time-scale capacity. Our goals here were to **(1)** isolate the thermogenic contribution of skeletal muscle from other potential thermogenic tissues following PO, and **(2)** capture the full length of the PO-induced thermogenesis. To achieve this, we measured gastrocnemius temperature via surgically implanted transponders at regular intervals until temperatures returned to baseline.

**Figure 1.**
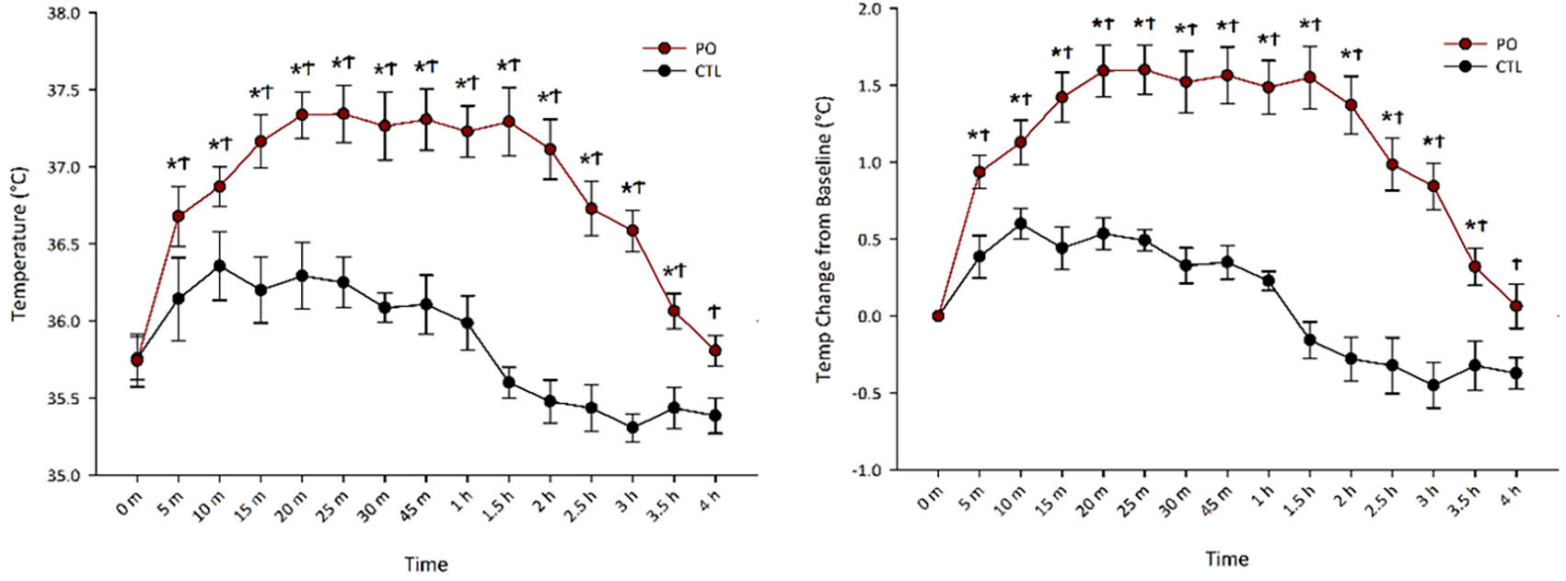
Predator odor-induced muscle thermogenesis lasts 4 hrs in rats. Gastrocnemius muscle temperature (A) and temperature (temp) change from baseline (B) across time following odor (PO) exposure. *Significant difference between baseline temperature and timepoint temperature within PO condition, *p* < 0.05; ϮSignificant difference between PO and control odor, *p* < 0.05; PO, predator odor; CTL, control odor.

Figure 1 shows that PO exposure rapidly increased gastrocnemius muscle thermogenesis, peaking at 20 min ( $\bar{X}$ = 1.60°C) and dissipating near baseline by 4 hr ( $\bar{X}$ = 0.06°C). A two-way repeated-measures ANOVA of raw temperature change showed a main effect of odor, F(1, 6) = 107.76, *p* < 0.001, where PO exposure resulted in higher muscle temperatures ( $\bar{X}$ = 36.84°C, SEM = 0.15°C) compared to exposure to control odor ( $\bar{X}$ = 35.86°C, SEM = 0.13°C). Follow-up t-tests indicated that PO significantly increased thermogenesis from baseline beginning 5 min after PO exposure and persisting for 3.5 h. There was also a significant interaction between odor and time, F(14, 84) = 11.99, *p* < 0.001, where PO increased thermogenesis more than control odor and the magnitude of this difference changed with time.

To control for individual differences in muscle temperature, we performed a two-way repeated measures ANOVA on temperature change from baseline which revealed the same pattern as raw temperature change with a main effect of odor, F(1, 6) = 93.08, *p* < 0.001, where PO ( $\bar{X}$ = 1.09°C, SEM = 0.10°C) increased muscle thermogenesis more than control odor ( $\bar{X}$ = 0.98°C, SEM = 0.05°C). Follow up t-tests indicated that PO significantly increased thermogenesis from baseline beginning 5 min after PO exposure and persisting for 3.5 h. There was also a significant interaction between odor and time, F(14, 84) = 11.99, *p* < 0.001, where PO increased thermogenesis more than control odor and the magnitude of this difference changed with time.

### 3.2 Predator odor exposure increases plasma corticosterone acutely

**Figure 2.**
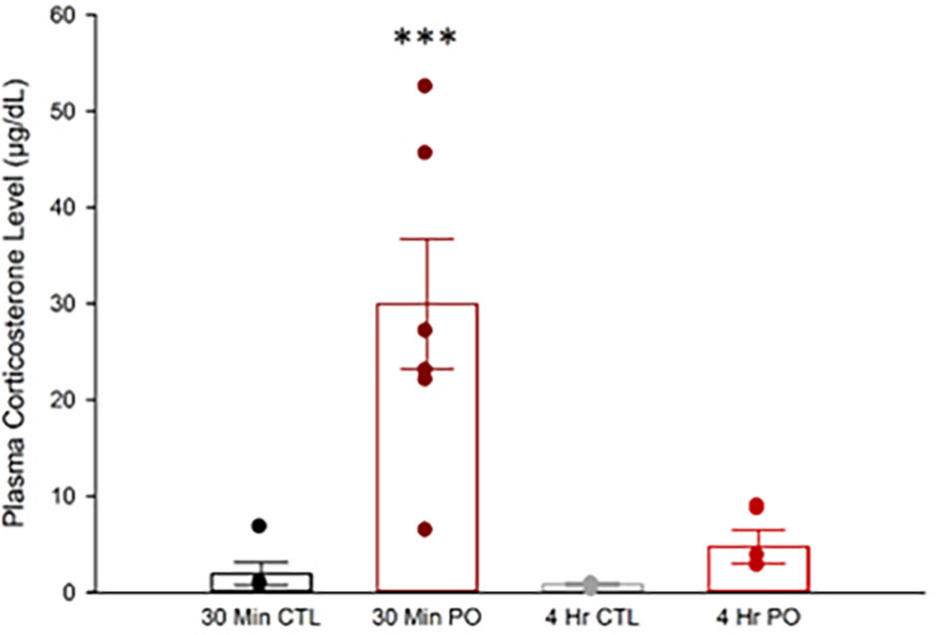
Predator odor exposure elevated circulating corticosterone in rats. Plasma corticosterone levels following either PO or control odor after either 30 min or 4 hr. ***Significant interaction between odor and time, *p* < 0.001; CTL, control odor; PO, predator odor.

Skeletal muscle is a major target of corticosterone where it can cause proteolysis to rapidly increase energy availability during psychologically stressful events ( Bodine and Furlow, 2015). Acute increases in corticosterone are beneficial, but chronic increases can cause muscle atrophy ( Tomas et al., 1979). Just 10 min of ferret exposure significantly increases plasma corticosterone levels which dissipate near control levels after 1 hr in rats ( Roseboom et al., 2007). However, it is unknown if corticosterone levels remain elevated following 4 hr of constant PO exposure. We found that PO exposure significantly increased plasma corticosterone after 30 min but not 4 hr ( Figure 2). A two-way ANOVA showed a significant interaction between time and odor, F(1, 20) = 11.55, *p* = 0.003, where PO ( $\bar{X}$ = 29.94 μg/dL, SEM = 3.55 μg/dL), but not control odor ( $\bar{X}$ = 1.95 μg/dL, SEM = 3.55 μg/dL), significantly increased plasma corticosterone after 30 min of exposure, and this difference ceased after 4 hr of exposure ( *p* > 0.05). This suggests that the animals engaged effective coping mechanisms and likely avoided corticosterone-induced muscle damage.

### 3.3 *Sirt1* and *Bdnf* expression in the vmh are increased following 4 hr of predator odor exposure

**Figure 3.**
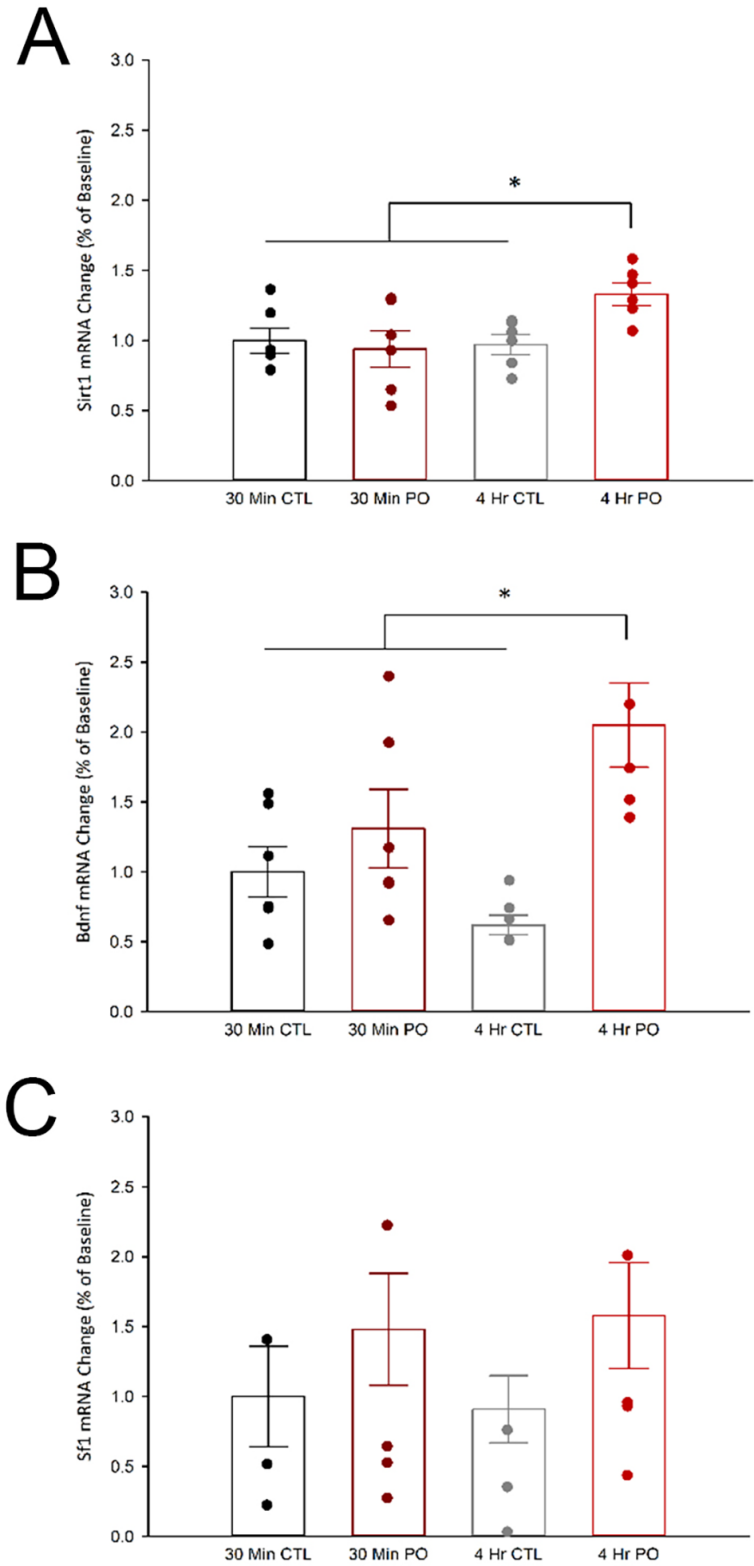
Predator odor exposure increased *Sirt1* and *BDNF*, but not *Sf1*, mRNA, after 4 hrs. **(A)** *Sirt1*, **(B)** *BDNF*, and **(C)** *Sf1* mRNA expression levels in the VMH following either PO or control odor exposure after either 30 min or 4 hr. *Significant interaction between odor and time, *p* < 0.05; CTL, control odor; PO, predator odor.

While several genes in VMH cells have been explored in the context of metabolism, to our knowledge, virtually none have been explored in the context of PO exposure. Metabolic studies have implicated VMH expression of SIRT1, BDNF, and SF-1 as possible mediators of skeletal muscle metabolism ( Wang et al., 2010; Ramadori et al., 2011; Kim et al., 2012). Here, we asked if the VMH transcription of these genes correlated with time points when PO-induced muscle thermogenesis peaks and returns-to-baseline. We found that PO exposure significantly increased *Sirt1* and *Bdnf* expression in the VMH after 4 hr but not 30 min ( Figure 3), where two-way ANOVAs showed a significant interactions between time and odor. Compared to control odor, PO exposure increased *Sirt1* mRNA expression after 4 hr, but not 30 min, F(1, 20) = 4.96, *p* = 0.03, and PO exposure increased *Bdnf* mRNA expression after 4 hr, but not 30 min, F(1, 20) = 6.15, *p* = 0.022. Unexpectedly, there were no significant changes in *Sf1* expression in the VMH following 30 minutes or 4 hours of PO exposure; two-way ANOVA results showed no significant changes in *Sf1* mRNA expression in any condition ( *p* < 0.05).

### 3.4 RNA-sequencing identifies transcriptome changes in the vmh following acute predator odor exposure

**Figure 4.**
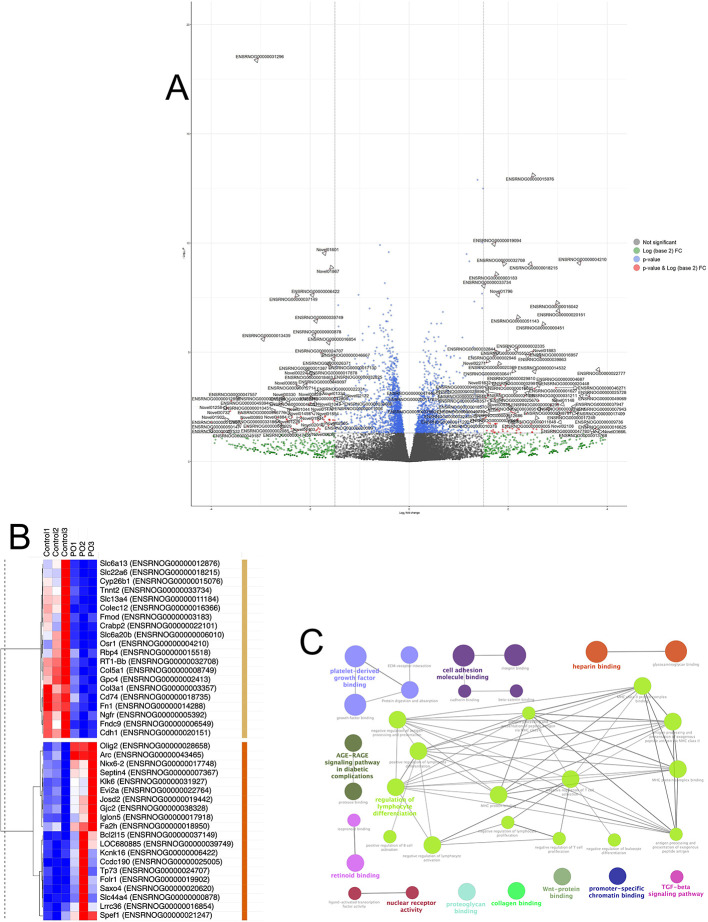
Transcriptome changes in the VMH following acute predator odor exposure. (A) Volcano plot of the differentially expressed genes between control and PO. Non-significant genes are shown in grey; blue and green indicate the gene expression was above one cut-off criterion but not the other (padj and fold change). Significant genes are shown in red, identified by their respective Ensembl IDs (see Extended Data Table 1 for the full list). Black vertical lines highlight log fold changes of –1.5 and 1.5, while black horizontal line represents a padj of 0.05. (B) Heatmap of the top 20 up- and down-regulated by PO genes (built using software.broadinstitute.org/morpheus/) where the color scheme reflects relative individual expression values for each gene, ranging from the minimum values in dark blue, to the maximum values in dark red. Hierarchical clustering of genes is based on 1-Pearson correlation values using average linkage. Clusters of genes up- and down-regulated by PO are marked with brown and yellow bars, respectively. (C) Functional enrichment networks of Gene Ontology Molecular Function, and Immune System Process terms and KEGG pathways of differentially expressed protein-coding genes, visualized using ClueGO Cytoscape plugin, with connectivity measure of kappa score ≥ 0.4 and enriched pathways p value padj ≤ 0.05. Functional groups are shown with different colors, with the terms marked in bold signifying the most essential pathway-defining terms for each major group (see Extended Data Table 2 for the full list).

Although the VMH is a known regulator of metabolic and predator threat responses, the cellular and molecular alterations following PO exposure are unknown. Thus, it is difficult to investigate how the VMH might regulate muscle metabolism following PO exposure. Here, we performed RNA-sequencing on VMH samples from animals who were exposed to either 20 min of PO or control odor exposure. Differential gene expression analysis revealed 164 DEGs. Of these DEGs, 60 genes were up-regulated and 103 genes were down-regulated. Table 2 shows the top 20 upregulated and top 20 downregulated DEGs. A GO enrichment analysis was performed using the R package GOseq to control for gene transcript length. Our GOseq analysis revealed 65 significantly enriched GO terms. We curated a list of the top 20 most enriched terms by removing GO terms that were 1) identical but with different accession numbers, 2) redundant “parent” terms, or 3) functionally irrelevant (e.g., endoderm development) ( Figure 4B). Our pathway enrichment analysis revealed 28 significantly enriched KEGG pathways ( Figure 4C).

**Table 2.** Top 20 up-regulated and down-regulated genes in the ventromedial hypothalamus following predator odor (PO) exposure.

Gene symbol	Ensembl gene ID	log2 fold change	padj	Gene description
***Down-regulated by PO***				
Slc13a4	ENSRNOG00000011184	2.43639	1.27E-20	solute carrier family 13 member 4 (Slc13a4)
Cyp26b1	ENSRNOG00000015076	2.48083	3.23E-10	cytochrome P450, family 26, subfamily b, polypeptide 1 (Cyp26b1)
Col3a1	ENSRNOG00000003357	1.38085	3.65E-10	collagen type III alpha 1 chain (Col3a1)
Slc6a20b	ENSRNOG00000006010	1.49351	7.50E-10	solute carrier family 6 member 20b (Slc6a20b)
Slc6a13	ENSRNOG00000012876	1.47612	1.71E-07	solute carrier family 6 member 13 (Slc6a13)
Ngfr	ENSRNOG00000005392	1.16095	3.78E-07	nerve growth factor receptor (Ngfr)
Cd74	ENSRNOG00000018735	1.21395	7.18E-07	CD74 molecule (Cd74)
Osr1	ENSRNOG00000004210	3.46218	7.18E-07	odd-skipped related transcription factor 1 (Osr1)
RT1-Bb	ENSRNOG00000032708	1.89288	7.30E-07	RT1 class II, locus Bb (RT1-Bb)
Slc22a6	ENSRNOG00000018215	2.47570	9.39E-07	solute carrier family 22 member 6 (Slc22a6)
Fmod	ENSRNOG00000003183	1.73685	2.41E-06	fibromodulin (Fmod)
Tnnt2	ENSRNOG00000033734	1.53081	5.60E-06	troponin T2, cardiac type (Tnnt2)
Col5a1	ENSRNOG00000008749	1.47249	1.29E-05	collagen type V alpha 1 chain (Col5a1)
Colec12	ENSRNOG00000016366	1.31716	2.49E-05	collectin sub-family member 12 (Colec12)
Crabp2	ENSRNOG00000022101	1.47596	4.36E-05	cellular retinoic acid binding protein 2 (Crabp2)
Fn1	ENSRNOG00000014288	0.68015	5.96E-05	fibronectin 1 (Fn1)
Cdh1	ENSRNOG00000020151	3.03569	5.98E-05	cadherin 1 (Cdh1)
Gpc4	ENSRNOG00000002413	0.64688	1.52E-04	glypican 4 (Gpc4)
Rbp4	ENSRNOG00000015518	1.07312	4.53E-04	retinol binding protein 4 (Rbp4)
Fndc9	ENSRNOG00000006549	0.73824	9.53E-04	fibronectin type III domain containing 9 (Fndc9)
***Up-regulated by PO***				
Olig2	ENSRNOG00000028658	-0.58890	1.94E-07	oligodendrocyte transcription factor 2 (Olig2)
Kcnk16	ENSRNOG00000006422	-1.92925	1.29E-05	potassium two pore domain channel subfamily K member 16 (Kcnk16)
Arc	ENSRNOG00000043465	-1.02244	1.40E-05	activity-regulated cytoskeleton-associated protein (Arc)
Bcl2l15	ENSRNOG00000037149	-2.29616	1.65E-05	Bcl2-like 15 (Bcl2l15)
Ccdc190	ENSRNOG00000025005	-1.43208	3.28E-05	coiled-coil domain containing 190 (Ccdc190)
Nkx6-2	ENSRNOG00000017748	-0.79974	4.37E-05	NK6 homeobox 2(Nkx6-2)
LOC680885	ENSRNOG00000039749	-1.85775	1.14E-04	hypothetical protein LOC680885(LOC680885)
Josd2	ENSRNOG00000019442	-0.58964	1.73E-04	Josephin domain containing 2 (Josd2)
Klk6	ENSRNOG00000031927	-0.81743	1.95E-04	kallikrein related-peptidase 6 (Klk6)
Folr1	ENSRNOG00000019902	-1.42678	1.95E-04	folate receptor alpha (Folr1)
Septin4	ENSRNOG00000007367	-0.58757	2.19E-04	septin 4 (Septin4)
Gjc2	ENSRNOG00000038328	-0.71012	3.30E-04	gap junction protein, gamma 2 (Gjc2)
Saxo4	ENSRNOG00000020620	-0.94092	3.40E-04	stabilizer of axonemal microtubules 4 (Saxo4)
Slc44a4	ENSRNOG00000000878	-1.89611	3.50E-04	solute carrier family 44, member 4 (Slc44a4)
Lrrc36	ENSRNOG00000016854	-1.60202	6.16E-04	leucine rich repeat containing 36 (Lrrc36)
Fa2h	ENSRNOG00000018950	-0.65593	1.08E-03	fatty acid 2-hydroxylase (Fa2h)
Iglon5	ENSRNOG00000017918	-0.68580	1.37E-03	IgLON family member 5 (Iglon5)
Tp73	ENSRNOG00000024707	-1.77753	1.57E-03	tumor protein p73 (Tp73)
Evi2a	ENSRNOG00000022764	-0.68777	1.64E-03	ecotropic viral integration site 2A (Evi2a)
Spef1	ENSRNOG00000021247	-0.60595	1.64E-03	sperm flagellar 1 (Spef1)

### 3.5 RNA-sequencing reveals alterations associated with synaptic plasticity

**Figure 5.**
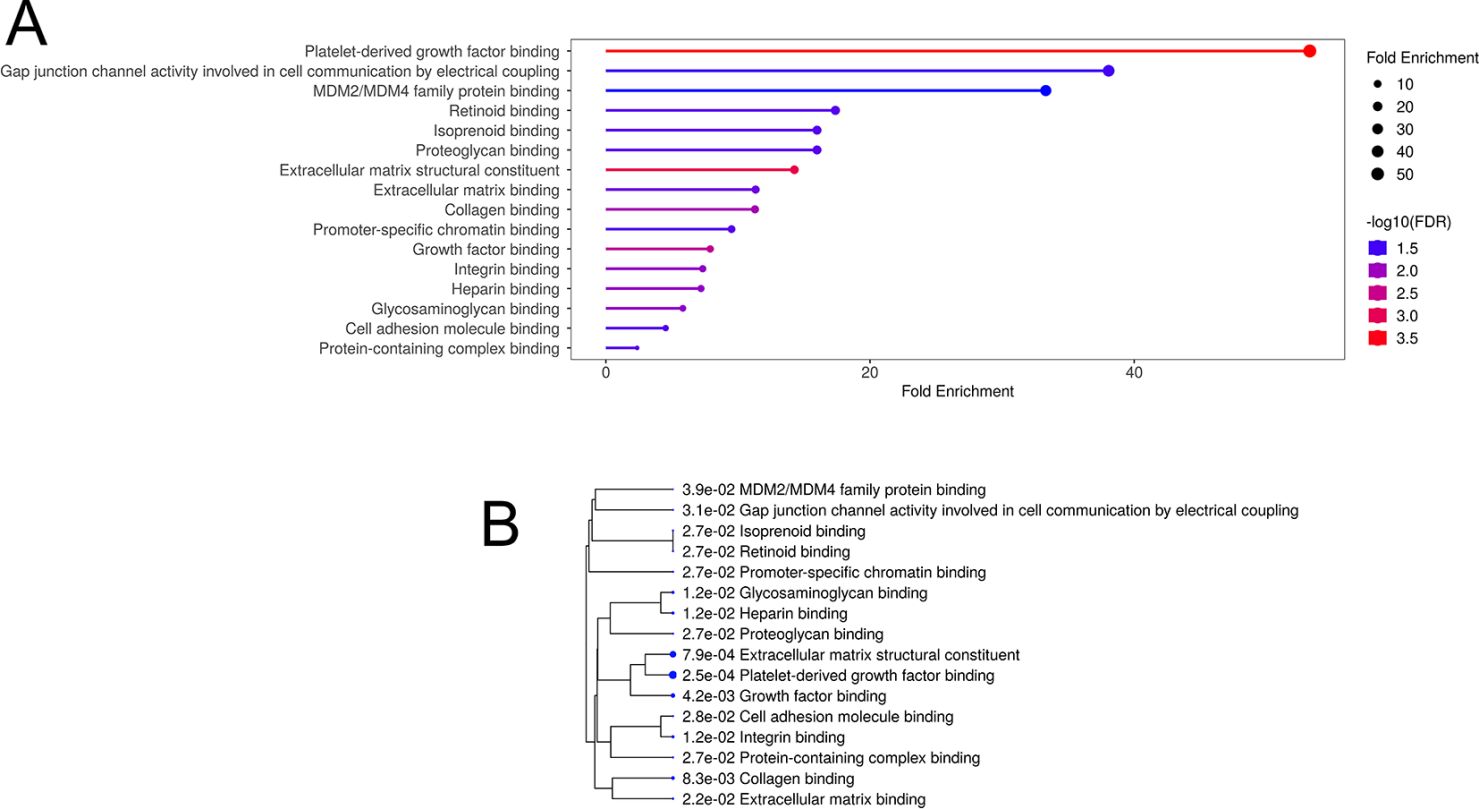
Over-enrichment (A) and hierarchical clustering (B) of significantly enriched Gene Ontology Molecular Function pathways, where pathways that share genes cluster together. Bigger dots indicate smaller p-values. Each pathway includes at least two genes, with FDR cut-off of 0.05.

To further explore whether identified DEGs relevant to synaptic plasticity, protein-coding DEGs were annotated using DAVID (The Database for Annotation, Visualization and Integrated Discovery, ver. v2023q3) ( Sherman et al., 2022), resulting in 47 genes annotated with GO terms related to keywords “synaptic”, “neural”, “nervous” or “neuron”. We further used ShinyGO 0.77 ( Ge et al., 2020) to identify 16 over-enriched GO terms with at least two genes per pathway ( Figure 5, Table S3A). Notably, all but two of these pathways (Retinoid binding and Isoprenoid binding) contained genes associated with nervous system functions and/or synaptic plasticity. Likewise, of the top 40 up- and down-regulated DEGs, 15 genes were annotated with nervous system-related terms (Table S3B). Moreover, among DEGs were eight neuronal immediate early genes (IEGs), namely Btg2, Fosb, Arc, Apold1, Egr1, Egr4, Nr4a1, Sgk1 (Table S1), of which Arc was one of the top 20 up-regulated DEGs. Arc, activity-regulated cytoskeleton-associated protein, translocates from the nucleus to cell dendrites in an activity dependent manner, where, after rapid translation, it interacts with F-actin to potentially modify dendritic spines ( Pinaud and Tremere, 2006). There are also many other DEGs with known involvement in synaptic plasticity, including Bcas1 (brain enriched myelin associated protein 1), Cdh1 (cadherin 1), multiple collagen chain genes, multiple members of the solute carrier family, Septin4, Syndig1 (synapse differentiation inducing 1), and Snap91 (synaptosome associated protein 91).

## 4. Discussion

The VMH is required for optimal regulation of energy balance and does so through its control of the autonomic nervous system and its complex pathways with other hypothalamic nuclei and brain regions ( Dhillon et al., 2006; King, 2006; Xu et al., 2010; Kim et al., 2011, 2012; Ramadori et al., 2011). The VMH also plays an important role in regulating metabolic and behavioral responses to predator threat, during which peripheral thermogenesis and EE are increased ( Dielenberg et al., 2001, 2004; Silva et al., 2013, 2016; Cheung et al., 2015; Kunwar et al., 2015; Viskaitis et al., 2017; Shemery et al., 2023). While this dual-processing role of the VMH has been well established, the cellular and molecular mechanisms involved in VMH metabolic control in the context of predator threat are not fully understood. Here, we sought to provide an initial exploration of gene expression changes within the VMH at time points associated with peak and returned-to-baseline skeletal muscle temperatures following PO exposure ( Figure 1). First, we established a temporal profile for VMH *Sirt1*, *Bdnf*, and *Sf1* expression following PO exposure, with increased expression of *Bdnf* and *Sirt1* after 4 hr of PO exposure but not 30 min. Interestingly, *Sf1* levels were not significantly altered following either 30 min or 4 hr of PO exposure. As predicted, PO exposure increased corticosterone levels after 30 min of PO exposure, but despite continued PO exposure, corticosterone levels decreased to control levels after 4 hrs. Next, RNA-sequencing identified 164 DEGs, 65 enriched GO terms and 33 enriched KEGG pathways. Altogether, these results reveal important themes of immune and oxidative stress responses, and synaptic plasticity in the VMH in response to predator threat.

### 4.1 Implications of the temporal profiles for *Sirt1*, *Bdnf*, and *Sf1*

*Sirt1*, *Bdnf*, and *Sf1* are metabolically responsive genes in the VMH implicated in the regulation of skeletal muscle metabolism. We have found that PO exposure consistently induces peak skeletal muscle thermogenesis within 15-30 min ( Gorrell et al., 2020), after which it gradually declines, reaching baseline at 4 hr ( Figure 1), thus we opted to measure *Sirt1*, *Bdnf*, and *Sf1* mRNA levels at these thermogenically relevant time points. Our results show that *Sirt1*, *Bdnf*, and *Sf1* were not significantly altered after 30 min of PO exposure ( Figure 3). This suggests that while VMH expression of these genes may modulate peripheral metabolism, their transcription may not be necessary for the immediate thermogenic response to PO exposure. Further, *Sirt1* and *Bdnf* mRNA levels were significantly increased following 4 hr of PO exposure, a time point associated with returned-to-baseline thermogenesis. Thus, VMH *Sirt1* and *Bdnf* may not be necessary for an acute induction of muscle thermogenesis, but instead have roles associated with the thermogenic or predator-stress outcomes. SIRT1 is a nicotinamide adenosine dinucleotide (NAD ^+^)-dependent histone deacetylase believed to have cell-protecting effects in response to metabolic changes and environmental stressors ( Chang and Guarente, 2014). However, there are conflicting reports on the benefits of SIRT1 expression in the hypothalamus. For example, Ramadori et al. (2011) and Cakir et al. (2009) assert that, following reduced energy availability, SIRT1 expression increases in the hypothalamus, whereas Sasaki et al. (2010) found the opposite. Because predator escape is energetically costly and we have shown heightened skeletal muscle thermogenesis that persists for nearly 4 hours ( Figure 1), it is possible that the delayed increase in *Sirt1* mRNA was a response to depleted energy availability secondary to the thermogenic processes. This would seem inconsistent with reports demonstrating that VMH SIRT1 expression increases insulin-stimulated glucose uptake in skeletal muscle and is associated with increased EE, though ( Ramadori et al., 2011; Aras et al., 2019). Because increased SIRT1 activity has been reported in the brain following acute PO exposure ( Yu et al., 2018), it is possible that the delayed increase in *Sirt1* mRNA observed here was a response to psychological stress. Interestingly, Libert et al. (2011) suggest that whole brain SIRT1 likely integrates metabolic and predatory information such that food scarcity and high predation are associated with high brain SIRT1, resulting in vigilance and reduced exploration. Due to the unique role of the VMH in processing predator threat and metabolic responses including skeletal muscle metabolism, the timing and function of SIRT1 expression may be complex.

Delayed increase in *Bdnf* mRNA following PO exposure was unexpected given that VMH BDNF microinjection immediately increases EE ( Wang et al., 2010) and a single glucose injection rapidly increases VMH BDNF expression ( Unger et al., 2007). It is possible that delayed increase in *Bdnf* mRNA may implicate its role in stress-induced synaptic plasticity. Glucocorticoids, including corticosterone, interact with BDNF in the brain to modify synapses and these interactions are associated with stress and depression resilience ( Tapia-Arancibia et al., 2004; Kunugi et al., 2012; Jeanneteau and Chao, 2013; Numakawa et al., 2013; Daskalakis et al., 2015). Not only have glucocorticoid-BDNF interactions been confirmed in the hypothalamus following stress, but BDNF in VMH SF-1 neurons modifies GABAergic inputs onto SF-1 cells ( Tapia-Arancibia et al., 2004; Jeanneteau et al., 2012; Jo, 2012). The data reported here data reveal an unexpected but novel finding regarding the temporal relationships among *Sirt1*, *Bdnf*, and *Sf1* mRNA expression in the VMH following acute PO exposure. While the expression of these genes may not be necessary for the immediate thermogenic response to a single PO exposure, they may have roles in regulating subsequent thermogenic or behavioral adaptation to PO.

### 4.2 Oxidative stress and immune responses

Inflammation and oxidative stress are distinct processes but are closely connected and can have a reciprocal relationship. Brain oxygen consumption is high, making it vulnerable to oxidative stress which can trigger inflammation ( Leszek et al., 2016). In turn, brain inflammation can induce oxidative stress ( Muriach et al., 2014). Our RNA-sequencing results suggest that acute PO exposure may rapidly alter immune and oxidative stress responses within the VMH. Indeed, predator threat alters oxidative stress and inflammatory responses in the brain, but no previous reports have specifically implicated VMH inflammation ( Toumi et al., 2013; Wilson et al., 2013; Mejia-Carmona et al., 2014, 2015). Mejia-Carmona et al. (2015) reported that recurrent PO exposure across 6 days led to increased manganese-dependent mitochondrial superoxide dismutase (MnSOD) in the hypothalamus, indicating that the hypothalamus engaged a protective antioxidant response to an early increase of superoxide radicals. The inflammatory effect of predator threat on the hypothalamus is less consistent. Plata-Salaman et al. (2000) found that neither acute nor repeated exposure to a ferret increased proinflammatory cytokines in rats, while Barnum et al. (2012) reported that acute exposure to an aggressive rat increased proinflammatory cytokines in mice for up to 8 hr. Regardless, prolonged or chronic predator threat is associated with post-traumatic stress disorder (PTSD) and accelerated aging, which are clinically intertwined with major health issues including cardiovascular disease, metabolic dysregulation, and dementia ( Oosthuizen et al., 2005; Epel, 2009; Wilson et al., 2013; Mellon et al., 2018).

Interestingly, PO exposure also alters expression of several genes in the KEGG Toxoplasmosis pathway ( Figure 4C). Changes in this pathway are intriguing given that infection with *Toxoplasmosa gondii* markedly attenuates rats’ behavioral response to predators ( Berdoy et al., 2000). Ultimately, our RNA-sequencing results reveal the potential for acute PO exposure to rapidly alter multiple stress-response pathways in the VMH. It will be critical to understand if or how these alterations are associated with the induction of skeletal muscle thermogenesis as well as behavioral responses to predator threat.

### 4.3 Synaptic plasticity

The DEGs and enriched pathways identified via RNA-seq suggest that acute PO exposure may alter synaptic plasticity in the VMH. Synaptic plasticity in the adult hypothalamus has been highly investigated since it was first observed in the 1980s ( Theodosis et al., 1981, 1986; Theodosis and Poulain, 1984, 1987; Tweedle and Hatton, 1984; Chapman et al., 1986). Hypothalamic synapses and their astrocytes undergo morphological changes in response to gestation ( Theodosis and Poulain, 1984), lactation, ( Theodosis et al., 1981, 1986; Theodosis and Poulain, 1984; Stern and Armstrong, 1998) and dehydration ( Tweedle and Hatton, 1984; Chapman et al., 1986; Miyata et al., 1994). Several hypothalamic nuclei, especially the VMH, undergo changes in dendritic arborization and spine density in response to gonadal hormones and sexual behavior ( Frankfurt et al., 1990; Parducz et al., 1993; Garcia-Segura et al., 1994a, 1994b; Xiong et al., 1997; Calizo and Flanagan-Cato, 2000; Calizo and Flanagan-Cato, 2002; Flanagan-Cato et al., 2006; Griffin and Flanagan-Cato, 2008). Importantly, hypothalamic plasticity is also critical for energy homeostasis ( Horvath et al., 1999; Bouret et al., 2004; Horvath and Diano, 2004; Horvath, 2005; Horvath and Gao, 2005; Andrews et al., 2008; Benani et al., 2012; Hu et al., 2014; Qi and Yang, 2015; Dodd et al., 2018). Feeding circuits within the arcuate nucleus (ARC) undergo rapid, plastic changes that consistently flip-flop in response to satiety and hunger signals ( Pinto et al., 2004; Yang et al., 2011). Interestingly, the VMH projects excitatory synaptic inputs to pro-opiomelanocortin neurons in the ARC that become diminished during fasting ( Sternson et al., 2005). Fasting also causes alterations in dendritic arborization and soma size within the VMH ( Flanagan-Cato et al., 2008). Taken together, these findings suggest that the VMH has the capacity for synaptic plasticity in response to behavioral states that it regulates as well as metabolic state.

Though not directly implicated, there is also evidence to suggest that the VMH likely undergoes synaptic plasticity following acute PO exposure. Silva et al. (2016) demonstrated that the VMH was necessary for the acquisition and expression of fear memory following a single, acute predator exposure ( Silva et al., 2016). Moreover, expression of a protein in the voltage-dependent calcium channel complex, Alpha2delta-1, in VMH SF-1 neurons controls excitatory synaptogenesis to modulate sympathetic activation of skeletal muscle ( Lau et al., 2016). This suggests that sympathetic activation induced by PO could result from rapid, plastic changes within the VMH, or to other brain regions directly innervated by VMH neuronal afferents. Indeed, VMH stimulation induces hippocampal plasticity ( Ying et al., 2012). Importantly, predator stress induces plasticity in a variety of brain regions ( Adamec et al., 1999, 2003; Li et al., 2004; Adamec et al., 2006; Thomas et al., 2006; Park et al., 2008; Zoladz et al., 2012; Mitra, 2019), including the hypothalamus ( Inoue et al., 2013). A single episode of acute PO exposure induces long-term potentiation at GABA synapses in another hypothalamic nucleus, the paraventricular nucleus of the hypothalamus, which may sensitize future stress responses because GABA synapses become excitatory during acute stress ( Inoue et al., 2013). It is also important to note that acute predator stress does not appear to alter the proliferation or survival of neurons, suggesting our plasticity-associated DEGs are likely mediating plasticity in existing neurons rather than neurogenic processes ( Lau et al., 2016). Thus, combined with our results, it seems highly probable that the VMH undergoes plasticity following acute PO exposure. This is not surprising given the critical survival value of avoiding food-seeking behavior during predator threat ( Viskaitis et al., 2017) as well as avoiding predation altogether ( Monarca et al., 2015).

### 4.4 Limitations

Examination of one time point limits the interpretation of our RNA-sequencing results. For example, reduced mRNA levels could stem from rapid translation of available transcripts before transcription is able to replenish the supply in which case, despite appearing downregulated, gene expression is important. Indeed, genes are transcribed and translated at variable rates depending on the gene, the tissue, the conditions, and new, mature mRNA can take up to an hour to be transcribed at noticeably altered levels ( Ressler et al., 2002; Sirri et al., 2010). Other limitations to our findings are the inherent flaws in RNA-sequencing ( Ozsolak and Milos, 2011; Zheng et al., 2011; Liu et al., 2014). RNA-sequencing lends itself to unavoidable biases due to technological limitations such as cDNA library construction artifacts, transcript mapping, read-count normalization, and GO analyses ( Ozsolak and Milos, 2011). Nonetheless, not only have major technological advances led to more reliable RNA-sequencing results, but the preservation of raw data allows for future re-analysis. Finally, the wide range of predator threat exposure protocols can hamper generalization or direct comparison to existing reports of PO-induced alterations in behavior, metabolism, or their underlying mechanisms.

In conclusion, these results are the first to reveal gene expression changes in the VMH following a single episode of PO exposure. Acute PO exposure rapidly modifies gene expression in the VMH at a time point that correlates with peak skeletal muscle thermogenesis, and implicates genes involved in immune and oxidative stress responses as well as synaptic plasticity. The transcription of *Sirt1* and *Bdnf*, genes implicated in skeletal muscle metabolism, are not significantly increased until 4 hr, when PO-induced thermogenesis resolves to baseline levels. Our data provide an excellent foundation from which we can guide exploration of VMH regulation of SNS activation following predator odor. Understanding these mechanisms could potentially lead to untangling the pathways that control stress and metabolic responses.

## Ethical considerations

Experiments involving rats were carried out with approval by the Kent State University Animal Care and Use Committee (approval number 460CN18-04) in accordance with the ethical guidelines provided in the Guide for the Care and Use of Laboratory Animals as well as the Association for Assessment and Accreditation of Laboratory Animal Care (AAALAC). Efforts were made to ameliorate suffering and minimize distress of animals using analgesics before and after surgical procedures, anesthesia using inhaled isoflurane (5% for induction, 2-3% for maintenance), and habituation prior to exposure to potentially stressful contexts.

## Data Availability

Repository: RNAseq data are deposited in NCBI GEO. Accession number: GSE142617; https://www.ncbi.nlm.nih.gov/geo/query/acc.cgi?acc=GSE142617. Open access software for statistical analysis can be found at https://www.r-project.org/. Tables S1-S3 are available through Figshare: ( Piontkivska et al., 2024) https://doi.org/10.6084/m9.figshare.25593420 **
Table S1.** Differentially expressed genes in response to predator odor. Columns H through M show DESeq2-normalized counts for control animals (H through J, and those exposed to predator odor, columns K through M). Gene symbol and description are per Biomart Ensembl annotations. 1 in column Q indicates which DEGs are protein-coding. **
Table S2.** Functional enrichment networks of Gene Ontology Molecular Function, and Immune System Process terms and KEGG pathways of differentially expressed protein-coding genes, visualized using ClueGO Cytoscape plugin, with connectivity measure of kappa score ≥ 0.4 and enriched pathways p value padj ≤ 0.05. Column L lists genes that are part of the respective GO categories and pathways listed in column B. **
Table S3A.** Enriched Gene Ontology pathways among differentially expressed genes in response to predator odor. Each pathway has at least two genes, with FDR cut-off of 0.05. Nervous-system related Gene Ontology term annotations among differentially expressed genes in response to predator odor. Per DAVID (The Database for Annotation, Visualization and Integrated Discovery, ver. v2023q3). doi: 10.6084/m9.figshare.25593420. Data are available under the terms of the Creative Commons Attribution 4.0 International license (CC-BY 4.0). Animal experiments adhere to ARRIVE guidelines. Repository: Figshare Project title: Supplementary Tables for Shemery, A., Gibson, M., Gorrell, E., Daniel, D., Piontkivska, H., and Novak, C.M., “RNA-sequencing Reveals Altered Gene Expression in the Ventromedial Hypothalamus Following Predator Odor Exposure”. ( Piontkivska et al., 2024). DOI: https://doi.org/10.6084/m9.figshare.25593420.v2 License: CC BY 4.0
