# Supplementary material for: RNA-sequencing Reveals Altered Gene Expression in the Ventromedial Hypothalamus Following Predator Odor Exposure
Source: F1000Res. 2025 Mar 5;13:648. Originally published 2024 Jun 17. [Version 2] doi: 10.12688/f1000research.152034.2 (PMC12648032; doi:10.12688/f1000research.152034.2)
Supplement: Peer review history

# Reviewer report: Reviewer response for version 2

**DOI:** 10.5256/f1000research.178331.r430387

**Published:** 2025-11-25

**Authors:** Li Li, Yanan Wu

**Affiliations:** 1 UT Southwestern Medical Center, Dallas, USA; 2 Department of Molecular Cellular and Developmental Biology, University of Michigan, Ann Arbor, michigan, USA

This paper investigates the molecular profiles of the ventromedial hypothalamus (VMH) in response to predator odor (PO)-induced non-exercise activity thermogenesis and during its recovery phase. The study utilizes physiological, endocrine, and transcriptomic analyses to explore how acute environmental stress influences gene expression and metabolic output (temperature, plasma CORT).

 The experimental design is thoughtful, and the questions asked are timely and well-defined. The work provides potentially important insight into how acute threat cues reshape VMH signaling to influence sympathetic outflow and muscle thermogenesis. The manuscript is overall well organized, though certain concepts and methodological details require clarification.

1. Framing the role of SF-1 more carefully. The current text suggests that SF-1 itself is involved in thermogenesis, therefore followed by the qPCR testing. However, the supporting evidence cited primarily shows that VMH SF-1-expressing neurons, rather than the SF-1 molecule per se, are key regulators of sympathetic outflow and skeletal muscle thermogenesis. Please consider a minor rewording, or cite more direct supporting studies (for example, Kim KW et al., 2011, PNAS).
2. Integrate molecular findings with physiological functions. The manuscript presents interesting molecular findings. The authors tried to tie them to physiological functions using GO terms and network analysis. Although commonly seen in bioinformatics, it somehow feels disconnected with the first half of the paper, where an interesting physiological question has already been presented, yet left unanswered. The manuscript would benefit from annotating specific gene clusters which might correspond to phases of thermogenic activation versus recovery, or metabolism in general; Or a certain gene reported in PO-induced freeze or fear reported elsewhere.
3. Sample size. Given the relatively small number of biological replicates, it would be helpful to acknowledge this limitation. And if possible, validate by qPCR in a separate cohort to reinforce key findings.

Minor comments

1. Method section 2.3: “…taking care to keep [t]he sample frozen throughout.”
2. Result section 3.4: please justify why different PO exposure durations were used across experiments (20min for sequencing v.s. 30min for temperature and CORT).

Is the work clearly and accurately presented and does it cite the current literature?

Yes

If applicable, is the statistical analysis and its interpretation appropriate?

Yes

Are all the source data underlying the results available to ensure full reproducibility?

Yes

Is the study design appropriate and is the work technically sound?

Yes

Are the conclusions drawn adequately supported by the results?

Partly

Are sufficient details of methods and analysis provided to allow replication by others?

Yes

Reviewer Expertise:

Thermogenesis, Hypothalamus

We confirm that we have read this submission and believe that we have an appropriate level of expertise to confirm that it is of an acceptable scientific standard, however we have significant reservations, as outlined above.

# Reviewer report: Reviewer response for version 2

**DOI:** 10.5256/f1000research.178331.r369666

**Published:** 2025-03-12

**Authors:** Yunhui Liu

**Affiliations:** 1 Shenzhen Institutes of Advanced Technology(SIAT), Chinese Academy of Sciences (CAS), Shenzhen, China

The authors have appropriately addressed the concerns I raised in my previous review and have made the necessary improvements to the study.

Is the work clearly and accurately presented and does it cite the current literature?

Partly

If applicable, is the statistical analysis and its interpretation appropriate?

Yes

Are all the source data underlying the results available to ensure full reproducibility?

Yes

Is the study design appropriate and is the work technically sound?

Yes

Are the conclusions drawn adequately supported by the results?

Partly

Are sufficient details of methods and analysis provided to allow replication by others?

Yes

Reviewer Expertise:

VMH，neural circuits，chronic stress, energy expenditure，bone metabolism

I confirm that I have read this submission and believe that I have an appropriate level of expertise to confirm that it is of an acceptable scientific standard.

# Reviewer report: Reviewer response for version 1

**DOI:** 10.5256/f1000research.166737.r295134

**Published:** 2024-07-18

**Authors:** Vito S Hernandez

**Affiliations:** 1 National Autonomous University of Mexico, Mexico, Mexico

The study investigates how predator odor (PO) exposure affects gene expression in the ventromedial hypothalamus (VMH) of rats. Given the convergence of functions of the VMH  in regulating body weight by modulating muscle metabolism and sympathetic nervous system (SNS) activity and in mediating behavioral responses to predator threats, I consider this an interesting and important study. The main objective was to uncover the molecular mechanisms behind VMH-mediated metabolic responses to predator threat.

 The authors evaluated transcriptomic changes after PO stress at two timepoints

 Exposure to PO caused a rapid increase in skeletal muscle thermogenesis, peaking at 30 minutes and returning to baseline by 4 hours. This was associated with increased energy expenditure 

**qPCR Results**: After 30 min (Peak termogenesis) no changes were observed in prescription of BdnF, Sirt1 or SF1. After 4 hours of PO exposure, there was an increased expression of Bdnf and Sirt1, but not SF1

**RNA-sequencing Results**: Identified 245 DEGs with at least a 1.5-fold change due to PO exposure.

 Functional Annotation: Overrepresented pathways among DEGs included immune response, oxidative stress, and synaptic plasticity.

 The findings indicate that acute PO exposure leads to both rapid and delayed changes in VMH gene expression, which likely have downstream metabolic consequences. These gene expression changes are linked to increased thermogenesis and energy expenditure, suggesting a complex interplay between stress response and metabolic regulation.

 Understanding the mechanisms by which predator odor can modify energy expenditure is a hot and very important topic. This study positions the VMH in the spotlight for further investigations. However the data obtained is to this point is correlational. No insight on the population of neurons that change their transcription in response to PO can be gained,  within the VMH/ can be obtained.

 The Peak thermogenic response is at 30 min , but the selected genes for PCR that were selected on a prior basis because their possible connection with modulating the EE in response to PO did not changed. The results form queue RNAseq showed on the other side some genes that were upregulated (though 1.5 times is a low threshold), but no clear correlation or discussion on the mechanisms by which changes in those genes can be important on the PO changes in EE is provided.

Is the work clearly and accurately presented and does it cite the current literature?

Yes

If applicable, is the statistical analysis and its interpretation appropriate?

I cannot comment. A qualified statistician is required.

Are all the source data underlying the results available to ensure full reproducibility?

Yes

Is the study design appropriate and is the work technically sound?

Yes

Are the conclusions drawn adequately supported by the results?

Partly

Are sufficient details of methods and analysis provided to allow replication by others?

Yes

Reviewer Expertise:

neuroendocrinology

I confirm that I have read this submission and believe that I have an appropriate level of expertise to confirm that it is of an acceptable scientific standard, however I have significant reservations, as outlined above.

# Response

**Published:** 2025-02-14

**Authors:** Colleen Novak

**Affiliations:** Dept Biological Sciences, Kent State University, Kent, Ohio, USA

***Reviewer 2***

*The study investigates how predator odor (PO) exposure affects gene expression in the ventromedial hypothalamus (VMH) of rats. Given the convergence of functions of the VMH  in regulating body weight by modulating muscle metabolism and sympathetic nervous system (SNS) activity and in mediating behavioral responses to predator threats, I consider this an interesting and important study. The main objective was to uncover the molecular mechanisms behind VMH-mediated metabolic responses to predator threat.*

*The authors evaluated transcriptomic changes after PO stress at two timepoints*

*Exposure to PO caused a rapid increase in skeletal muscle thermogenesis, peaking at 30 minutes and returning to baseline by 4 hours. This was associated with increased energy expenditure*

***qPCR Results**: After 30 min (Peak termogenesis) no changes were observed in prescription of BdnF, Sirt1 or SF1. After 4 hours of PO exposure, there was an increased expression of Bdnf and Sirt1, but not SF1*

***RNA-sequencing Results**: Identified 245 DEGs with at least a 1.5-fold change due to PO exposure.*

*Functional Annotation: Overrepresented pathways among DEGs included immune response, oxidative stress, and synaptic plasticity.*

*The findings indicate that acute PO exposure leads to both rapid and delayed changes in VMH gene expression, which likely have downstream metabolic consequences. These gene expression changes are linked to increased thermogenesis and energy expenditure, suggesting a complex interplay between stress response and metabolic regulation.*

*Understanding the mechanisms by which predator odor can modify energy expenditure is a hot and very important topic. This study positions the VMH in the spotlight for further investigations. However the data obtained is to this point is correlational. No insight on the population of neurons that change their transcription in response to PO can be gained,  within the VMH/ can be obtained.*

*The Peak thermogenic response is at 30 min , but the selected genes for PCR that were selected on a prior basis because their possible connection with modulating the EE in response to PO did not changed. The results form queue RNAseq showed on the other side some genes that were upregulated (though 1.5 times is a low threshold), but no clear correlation or discussion on the mechanisms by which changes in those genes can be important on the PO changes in EE is provided.*

**These considerations have been included in the revised discussion sections 4.1 and 4.4. Briefly, alterations in SF-1, BDNF, and SIRT1 have the potential to alter sympathetic nervous system outflow and thereby modulate muscle thermogenesis.**

# Reviewer report: Reviewer response for version 1

**DOI:** 10.5256/f1000research.166737.r295126

**Published:** 2024-07-10

**Authors:** Yunhui Liu

**Affiliations:** 1 Shenzhen Institutes of Advanced Technology(SIAT), Chinese Academy of Sciences (CAS), Shenzhen, China

The ventromedial hypothalamus (VMH) is crucial for maintaining energy balance by regulating autonomic nervous system activity and interacting with brain regions involved in feeding and thermoregulation. Additionally, it mediates diverse behaviors such as aggression, fear responses, memories, and aversion to predator odor. Acting as a dual processor for metabolic and predator threat responses, the VMH likely facilitates predator odor-induced skeletal muscle thermogenesis, yet the underlying cellular and molecular mechanisms remain unexplored.

 In this study, Shemery and colleagues used RNA sequencing to examine gene expression dynamics in the rat VMH following exposure to predator odor (PO), aiming to uncover its metabolic regulatory mechanisms in response to threats. They observed that brief PO exposure rapidly increased skeletal muscle thermogenesis, peaking at 20-30 minutes and returning to baseline after 4 hours. Using qPCR and RNA sequencing, they identified VMH genes that rapidly changed after PO exposure, implicating roles in immune responses, oxidative stress, and neural plasticity. Notably, genes like Sirt1 and Bdnf showed significant upregulation 4 hours post-exposure, suggesting their involvement in later-stage sympathetic nervous system (SNS) regulation under predator stress. This study provides an early glimpse into VMH gene expression changes following PO exposure, offering novel insights into its role in SNS regulation. While the authors posed high-quality scientific questions, their findings did not fully address them. For example, after brief PO exposure, they did not find any changes in mRNA related to metabolic regulation in the VMH. Below are the reviewer's suggestions for revisions:

 Major

 1）Although previous research by the authors showed that activation of melanocortin receptors in the VMH induces skeletal muscle thermogenesis (Gavini, 2016), there is still no direct evidence linking the VMH to the regulation of predator odor-induced skeletal muscle thermogenesis. Therefore, the authors should first establish the VMH's role in this process in the current study. It is recommended that they use in vivo calcium fluorescence recording with endoscopes or optical fibers to dynamically monitor VMH neuronal activity and analyze its correlation with the time course of predator odor-induced skeletal muscle thermogenesis.

 2）The authors chose BDNF, SIRT1, and SF-1 as candidate genes for qPCR to study the VMH's role in PO-induced skeletal muscle thermogenesis. However, the VMH also expresses many other receptors closely related to metabolism, such as insulin receptors (IR), leptin receptors (LepR), and melanocortin receptors (MR). The authors should expand their focus and thoroughly evaluate the role of these receptors in the VMH during PO-induced skeletal muscle thermogenesis.

 3）The reviewer has concerns about the VMH sampling process from rat brain slices. Given that the core data of this study were obtained through RNA sequencing and the VMH is very small, any deviation in sampling could introduce significant data bias. The authors should provide more details about the VMH extraction procedure.

 4）The authors' interpretation of the RNA sequencing results for the VMH is overly brief. They barely mention immune and oxidative stress responses in the Results section, yet describe these responses as equally important as synaptic plasticity in the Conclusion. The authors should provide a detailed analysis of the RNA changes related to immune and oxidative stress responses in the VMH following PO exposure in the Results section.

 Minor

 1) In the Abstract, the first sentence of the Methods and Results section, "Exposing rats to predator odor (PO) exposure causes a rapid increase in skeletal muscle thermogenesis…" contains an extra "exposure."

 2) Despite its small size, the VMH plays complex roles in metabolism, emotional regulation, mating behavior, and aggression. For instance, the dm/cVMH is closely linked to bone and energy metabolism abnormalities induced by chronic stress (Yang F et al., J Clin Invest. 2020, Shao J et al., Mol Psychiatry. 2022). Conversely, the vlVMH mediates the regulation of aggressive behavior. Additionally, the David J. Anderson group at the California Institute of Technology has conducted single-cell RNA sequencing on the vlVMH (Kim DW et al., Cell. 2019). The authors should compare their findings with existing studies in this field.

 3) Some of the literature cited in the manuscript doesn't adequately back up the authors' arguments, including but not limited to: a) In the third paragraph of the Introduction, the authors claim, "Importantly, increased VMH SF-1 expression improves skeletal muscle metabolism and is associated with increased SNS activation (Kim et al., 2012)." However, the cited paper does not establish a causal relationship between SF-1 expression and skeletal muscle metabolism. b) In the same paragraph, the authors state, "However, although PO induces c-fos in the VMH (Masini et al., 2005)…", but the original paper only found increased c-fos mRNA expression in the paraventricular nucleus (PVN) of the hypothalamus following PO stimulation. The expression of c-fos in the VMH was weak, as shown in Figure 2 of the original paper. The authors should verify their sources carefully and cite appropriate papers to support their key points accurately.

 4) In the methods and results section of the Abstract, the authors state, "This thermogenic response is associated with weight loss and increased EE even when controlling for physical activity." However, there is no corresponding source for this claim in the study, as they did not monitor weight, EE, or physical activity.

Is the work clearly and accurately presented and does it cite the current literature?

Partly

If applicable, is the statistical analysis and its interpretation appropriate?

Yes

Are all the source data underlying the results available to ensure full reproducibility?

Yes

Is the study design appropriate and is the work technically sound?

Yes

Are the conclusions drawn adequately supported by the results?

Partly

Are sufficient details of methods and analysis provided to allow replication by others?

Yes

Reviewer Expertise:

VMH，neural circuits，chronic stress, energy expenditure，bone metabolism

I confirm that I have read this submission and believe that I have an appropriate level of expertise to confirm that it is of an acceptable scientific standard, however I have significant reservations, as outlined above.

## References

[1] : A GABAergic neural circuit in the ventromedial hypothalamus mediates chronic stress-induced bone loss. *J Clin Invest* .2020;130(12) : 10.1172/JCI136105 6539-6554 10.1172/JCI136105 32910804 PMC7685749

[2] : Cav3.1-driven bursting firing in ventromedial hypothalamic neurons exerts dual control of anxiety-like behavior and energy expenditure. *Mol Psychiatry* .2022;27(6) : 10.1038/s41380-022-01513-x 2901-2913 10.1038/s41380-022-01513-x 35318460 PMC9156408

[3] : Multimodal Analysis of Cell Types in a Hypothalamic Node Controlling Social Behavior. *Cell* .2019;179(3) : 10.1016/j.cell.2019.09.020 713-728.e17 10.1016/j.cell.2019.09.020 31626771 PMC7534821

[4] : Ventromedial hypothalamic melanocortin receptor activation: regulation of activity energy expenditure and skeletal muscle thermogenesis. *J Physiol* .2016;594(18) : 10.1113/JP272352 5285-301 10.1113/JP272352 27126579 PMC5023712

[5] : Ferret odor as a processive stress model in rats: neurochemical, behavioral, and endocrine evidence. *Behav Neurosci* .2005;119(1) : 10.1037/0735-7044.119.1.280 280-92 10.1037/0735-7044.119.1.280 15727532 PMC2430889

# Response

**Published:** 2025-02-14

**Authors:** Colleen Novak

**Affiliations:** Dept Biological Sciences, Kent State University, Kent, Ohio, USA

***Reviewer 1, Major***

*1) Although previous research by the authors showed that activation of melanocortin receptors in the VMH induces skeletal muscle thermogenesis (Gavini, 2016), there is still no direct evidence linking the VMH to the regulation of predator odor-induced skeletal muscle thermogenesis. Therefore, the authors should first establish the VMH's role in this process in the current study. It is recommended that they use in vivo calcium fluorescence recording with endoscopes or optical fibers to dynamically monitor VMH neuronal activity and analyze its correlation with the time course of predator odor-induced skeletal muscle thermogenesis.*

**Author Response: **We have recently published data that links increased activity of VMH SF1 cells to muscle thermogenesis (Watts et al, 2024); this is now cited in the last paragraph of the introduction. As suggested by reviewer 2, fiber photometry could be employed to link VMH activity with SF1 neuronal activity, adding to what is already known regarding the activation of VMH neuronal populations upon predator-odor exposure. Unfortunately, unlike behaviors, a clear cessation of thermogenesis can’t be detected in muscle after temperature is elevated using current methods, which would hamper the correlation of neuronal activity with the time course of thermogenesis.

*2* *）* *The authors chose BDNF, SIRT1, and SF-1 as candidate genes for qPCR to study the VMH's role in PO-induced skeletal muscle thermogenesis. However, the VMH also expresses many other receptors closely related to metabolism, such as insulin receptors (IR), leptin receptors (LepR), and melanocortin receptors (MR). The authors should expand their focus and thoroughly evaluate the role of these receptors in the VMH during PO-induced skeletal muscle thermogenesis.*

**Author Response: **While we agree with the reviewer’s assessment, we were limited by the amount of RNA yielded by the micropunches, so we prioritized the targets most likely to be involved in both metabolic regulation and the response to predator threat. The reviewer’s point is acknowledged at the end of section 4.1 of the discussion in the context of informing how predator odor interacts with metabolism.

*3* *）* *The reviewer has concerns about the VMH sampling process from rat brain slices. Given that the core data of this study were obtained through RNA sequencing and the VMH is very small, any deviation in sampling could introduce significant data bias. The authors should provide more details about the VMH extraction procedure.*

**Author Response: **This procedure was adopted to avoid freeze-thaw cycles. This is now described in section 2.3 of the methods section. Ideally, methods like laser-capture microdissection, single-cell RNAseq, or RNA scope would allow for much greater neuroanatomical precision.

*4* *）* *The authors' interpretation of the RNA sequencing results for the VMH is overly brief. They barely mention immune and oxidative stress responses in the Results section, yet describe these responses as equally important as synaptic plasticity in the Conclusion. The authors should provide a detailed analysis of the RNA changes related to immune and oxidative stress responses in the VMH following PO exposure in the Results section.*

**Author Response: **We expanded the description of the analyses of DEGs related to the immune/inflammation and oxidative stress responses, in revised sections 3.4 and 3.5 of the results.

***Minor***

*1) In the Abstract, the first sentence of the Methods and Results section, "Exposing rats to predator odor (PO) exposure causes a rapid increase in skeletal muscle thermogenesis…" contains an extra "exposure."*

**Author Response: **Thank you for catching this typo!

*2) Despite its small size, the VMH plays complex roles in metabolism, emotional regulation, mating behavior, and aggression. For instance, the dm/cVMH is closely linked to bone and energy metabolism abnormalities induced by chronic stress (Yang F et al., J Clin Invest. 2020, Shao J et al., Mol Psychiatry. 2022). Conversely, the vlVMH mediates the regulation of aggressive behavior. Additionally, the David J. Anderson group at the California Institute of Technology has conducted single-cell RNA sequencing on the vlVMH (Kim DW et al., Cell. 2019). The authors should compare their findings with existing studies in this field.*

**Author Response: **A new paragraph has been added integrating this information with the current study at the end of section 4.3 of the discussion. We appreciate that you pointed out this work and its relevance to our paper.

*3) Some of the literature cited in the manuscript doesn't adequately back up the authors' arguments, including but not limited to: a) In the third paragraph of the Introduction, the authors claim, "Importantly, increased VMH SF-1 expression improves skeletal muscle metabolism and is associated with increased SNS activation (Kim et al., 2012)." However, the cited paper does not establish a causal relationship between SF-1 expression and skeletal muscle metabolism. b) In the same paragraph, the authors state, "However, although PO induces c-fos in the VMH (Masini et al., 2005)…", but the original paper only found increased c-fos mRNA expression in the paraventricular nucleus (PVN) of the hypothalamus following PO stimulation. The expression of c-fos in the VMH was weak, as shown in Figure 2 of the original paper. The authors should verify their sources carefully and cite appropriate papers to support their key points accurately.*

**Author Response: **This section has been edited to accurately reflect the evidence presented in the corrected references.

*4) In the methods and results section of the Abstract, the authors state, "This thermogenic response is associated with weight loss and increased EE even when controlling for physical activity." However, there is no corresponding source for this claim in the study, as they did not monitor weight, EE, or physical activity*.

**Author Response: **This sentence has been moved to the background section of the abstract since it refers to prior work.
